# Supplementary material for: E-cigarette-induced pulmonary inflammation and dysregulated repair are mediated by nAChR α7 receptor: role of nAChR α7 in SARS-CoV-2 Covid-19 ACE2 receptor regulation
Source: Respir Res. 2020 Jun 18;21:154. doi: 10.1186/s12931-020-01396-y (PMC7301079; doi:10.1186/s12931-020-01396-y)
Supplement: Supplementary file 1 — Additional file 1 Figure S1. Sub-chronic e-cig with PG + Nic exposure increased cotinine levels in mouse serum. Mice were exposed to e-cig with or without nicotine for 30 days, and blood was collected through submandibular venipuncture following the final exposure. Cotinine levels were measured in serum samples from WT and nAChR α7 KO mice exposed to PG or PG with nicotine by ELISA (Cat# CO096D, Calbiotech), (n = 3–10/group). Figure S2. Sub-chronic e-cig exposure decreased exercise capacity in nAChR α7 KO mice. Mice were exposed to e-cig with or without nicotine for 30 days, and treadmill for exercise ability measurements were performed before the day of sacrifice, after the last exposure. The running distance and running time for nAChR α7 global KO and WT mice (A and B, respectively) and nAChR α7 lung epithelial cell-specific floxed/Cre-CC10 KO mice (C and D, respectively) were recorded separately. (n = 3–6/group), (*P < 0.05 ***P < 0.001 between groups). Figure S3. Volcano plots represent the differentially expressed myeloid genes among air, PG with and without nicotine exposed WT and nAChR α7 KO mice. Nanostring mRNA data set obtained from Nanostring Technologies and analyzed using nSolver. (A) Comparisons between Air vs PG, Air vs PG + Nic, and PG vs PG + Nic in WT mice, with P < 0.05 considered a significant difference. (B) Comparison between WT and nAChR α7 KO mice exposed to air, PG or PG + Nic that will specifically determine the genotype-dependent change in expression of myeloid genes following e-cig exposure. Figure S4. Sub-chronic e-cig exposure induced pro-inflammatory mediators in BALF of nAChRα7 lung epithelial cell-specific KO mice. Bio-Plex Pro mouse cytokine 23-plex assay kit (Bio-Rad) was used to determine levels of pro-inflammatory cytokines/chemokines in BALF from nAChRα7 lung epithelial cell-specific KO mice exposed to e-cig for 30 days (2 h/day). Significant changes were found in pro-inflammatory cytokines IL-5, MCP-1, KC, Eotaxin, GM-CSF, and [file 12931_2020_1396_MOESM1_ESM.docx]

**Supplementary Figures**

Figure S1. **Sub-chronic e-cig with PG+Nic exposure increased cotinine levels in mouse serum.** Mice were exposed to e-cig with or without nicotine for 30 days, and blood was collected through submandibular venipuncture following the final exposure. Cotinine levels were measured in serum samples from WT and nAChR α7 KO mice exposed to PG or PG with nicotine by ELISA (Cat# CO096D, Calbiotech). (n= 3-10/group)

Figure S2. **Sub-chronic e-cig exposure decreased exercise capacity in nAChR α7 KO mice.** Mice were exposed to e-cig with or without nicotine for 30 days, and treadmill for exercise ability measurements were performed before the day of sacrifice, after the last exposure. The running distance and running time for nAChR α7 global KO and WT mice (A and B, respectively) and nAChR α7 lung epithelial cell-specific floxed/Cre-CC10 KO mice (C and D, respectively) were recorded separately. (n= 3-6/group). (*P<0.05. ***P<0.001 between groups).

Figure S3. **Volcano plots represent the differentially expressed myeloid genes among air, PG with and without nicotine exposed WT and nAChR α7 KO mice.** Nanostring mRNA data set obtained from Nanostring Technologies and analyzed using nSolver. (A) Comparisons between Air vs PG, Air vs PG+Nic, and PG vs PG+Nic in WT mice, with P <0.05 considered a significant difference. (B) Comparison between WT and nAChR α7 KO mice exposed to air, PG or PG+Nic that will specifically determine the genotype-dependent change in expression of myeloid genes following e-cig exposure.

Figure S4 **Sub-chronic e-cig exposure induced pro-inflammatory mediators in BALF of nAChRα7 lung epithelial cell-specific KO mice.** Bio-Plex Pro mouse cytokine 23-plex assay kit (Bio-Rad) was used to determine levels of pro-inflammatory cytokines/chemokines in BALF from nAChRα7 lung epithelial cell-specific KO mice exposed to e-cig for 30 days (2 hrs/day). Significant changes were found in pro-inflammatory cytokines IL-5, MCP-1, KC, Eotaxin, GM-CSF, and G-CSF (A). All the other pro-inflammatory cytokines such as IL-1α, TNFα, MIP-1β, IL-2, IL-9, IFNγ, RANTES, IL-6, IL-12p70, IL-13, IL-1β, Eotaxin, IL-3, IL-4, IL-10, IL-12p40, IL-17A, and MIP-1α (B and C) did not show any significant changes between the groups. Data are shown as mean ± SEM (n=3-5/group; equal number of male and female mice). (* P < 0.05, compared with air control).
